# Effective directional self-gathering of drops on spine of cactus with splayed capillary arrays

**DOI:** 10.1038/srep17757

**Published:** 2015-12-07

**Authors:** Chengcheng Liu, Yan Xue, Yuan Chen, Yongmei Zheng

**Affiliations:** 1 Key Laboratory of Bio-Inspired Smart Interfacial Science and Technology of Ministry of Education, School of Chemistry and Environment, Beihang University, Beijing, 100191 (P.R. China)

## Abstract

We report that the fast droplet transport without additional energy expenditure can be achieved on the spine of cactus (*Gymnocalycium baldianum*) with the assistance of its special surface structure: the cactus spine exhibits a cone-like structure covered with tilted scales. A single scale and the spine surface under it cooperatively construct a splayed capillary tube. The arrays of capillary tube formed by the overlapping scales build up the out layer of the spine. The serial drops would be driven by the asymmetric structure resulted from tilt-up scales-by-scales on the cone-shaped spine, and move directionally toward the bottom from top of spine, by means of the Laplace pressure in differences. In addition, after the past of the first droplet, thin liquid film of drop is trapped in the splayed capillary micro-tube on the surface of spine, which greatly reduces the friction of subsequential droplet transport in efficiency. This finding provides a new biological model which could be used to transport droplet spontaneously and directionally. Also this work offers a way to reduce the surface adhesion by constructing liquid film on the surface, which has great significance in prompting droplet transport efficiency.

Biological kingdom offers the strategies of liquid dynamics that adapt to the natural environment^1^,^2^,^3^,^4^,^5^,^6^,^7^,^8^, e.g., fog water collection controlled on biological surfaces such as spider silk^1^, beetle back^2^, plant leaf^3^, etc. in mist. They help researchers to understand the liquid behaviors^1^,^4^,^8^,^9^,^10^,^11^,^12^,^13^,^14^,^15^,^16^,^17^,^18^, and offers theories to design materials used for droplet transport including surfaces with asymmetric structure^1^,^3^,^4^,^7^, gradient wettability^5^ and additive energy to the systems^9^,^10^, etc. Up to now, varieties of materials and theories have been investigated to solve the problems in water acquiring task^9^,^19^,^20^,^21^,^22^,^23^,^24^,^25^. Cactuses are famous for their abilities to live in the desert where drought is normal and water evaporates so quickly. As has been reported, cactus spine covered with oriented spine and gradient grooves could drive droplet directionally^3^. Recently, we find that the scale-covered spines of cactus g*ymnocalycium baldianum* (lives in the desert area of Argentina), the structure of which is totally different from the reported one, could also propel fog drops directionally with the assistance of the tilted scales and the splayed capillary tubes. In addition, the subsequent droplets could move more quickly than the first one because the surface adhesion has been greatly reduced by the thin liquid film trapped in the splayed capillary tubes after the past of the first droplet. Our finding could provide researchers with useful guidelines for designing new materials used for drop transport, and it also provides researchers with a new way to prompt the droplet transport efficiency.

Here, we show the evolved spines of cactus g*ymnocalycium baldianum* (Fig. 1a), which is observed that the drops are self-transported directionally in mist (Fig. 1b) to achieve water collection in efficiency. The environmental scanning electron microscopy (ESEM) is used to observe the microstructure on spine of cactus g*ymnocalycium baldianum* (Fig. 1c). It is found that there are tilted scales overlapping along the length of the cone spine (~5 cm). The cone-apex angle *δ* of the spine is ~15° and the tilted angle of the scale (*α*) is ~20° to baseline of the axis of the spine (Fig. 1c). The scales are ~100–130 μm in length and ~30–50 μm in width (Fig. 1d). Under the tilted-up scales to base of spine, there are some cavities that can fan out with the maximum radius (*R*_*c*_) of ~20 μm (Fig. 1e) at the opens of scales and the minimum radius (*r*_*c*_) of ~8 μm at the ends of scales (Fig. 1f). The overlapping scales and the surface of the spine construct the gradient splayed capillary micro-tubes array along the cone-shaped spine of cactus (Fig. 1g).

As has been reported, cactus spines can transport fog droplets directionally^3^. In our experiment, although the structure of the scale-covered cactus spine is totally different from the reported one, it could also transport fog droplets directionally, which reveals the scale-covered spine may have different droplet transport mechanism arising from its special structure. The directional transport of fog drop is observed apparently as shown in Fig. 2a (also see Supplementary Video 1). In water condensation, drop 1′ formed on the tip of the spine and moved to the bottom of the spine (from ~1 s to ~20 s). Then drop 2′ formed on the spine and coalesced with drop 1′ (from ~40 s to ~80 s). With the passing of time, drop 3′ and drop 4′ formed successively and all moved to the bottom of the spine (from ~100 s to ~120 s). In whole process, all of the fog drops moved directionally, which exhibit effective self-gathering capacity along the spine. It is estimated that the average moving velocities of drops 3′ (formed at the time of ~100 s) and 4′ (formed at the time of ~120 s) were ~0.25 mm/s and ~0.27 mm/s, respectively. Both velocities were larger than the average moving velocity of drop 1′ (i.e., ~0.05 mm/s) (Fig. 2b). In addition, on tilted spine, fog drops can also be transported directionally (see Fig. S1).

To reveal the unique behaviors of drops on the spine, a drop in volume of 2 μl is used to mimic the fog drop because of the growing fog drop on fibers similar to the advancing behaviors of the deposited drop^26^. Firstly, the drop deposits on the tip of the spine (Fig. 3a). At the beginning, the drop spreads slightly (Fig. 3a, from 0 s to ~20 s) and then climbs up the first scale and expands in shape. Subsequently it moves near to the second scale quickly, and then climbs up the second scale and expands, and then covers the third scale (Supplementary Video 2). After passing through scale by scale, the drop moves finally to the bottom of spine (Fig. 3a, from ~20 s to ~160 s). Further observation is focused on solid-liquid interfaces in details as drop moves in time of ~20.20–20.27 s. It is found the liquid meniscus at the right side of droplet seemed to be dragged to the left and its curvature changed not obviously during the droplet motion (as indicated in the right-side images of Fig. 3b). In contrast, the curvature change of liquid meniscus at the left side of droplet (as indicated in the left-side images of Fig. 3b) was relatively obvious: at the beginning it spread slowly from scale 1 to scale 2 (from ~20.20 s to ~20.25 s). The curvature of the liquid meniscus between scale 1 and scale 2 grew gradually and finally reached the maximum (at the time of ~20.26 s). Then the liquid covered scale 2 and its curvature became relatively small in a short time (from ~20.26 s to ~20.27 s), whereby the entire droplet moved for a distance about the length of a single scale. In addition, the contacting angles measurements (see Fig. S2) during the droplet motion reveals there is no obvious difference between the contacting angles of the left and right sides of the drop. This result excludes the influence of the cone structure of the spine on droplet motion^5^. It also predicts the tilted scales on the spine surface plays an important role in the directional droplet motion.

Aimed to find out whether the tilted scale plays the key role in the droplet transport process, we performed the following controlling experiments. We deposited droplets with the fixed volume of 2 μl on spines with different surface structures: scale-off spine and normal groove spine of cactus (the structures are shown in Fig. S3–4). The drop placed on scale-covered spine spread asymmetrically (Fig. S5a). The droplets placed scale-cleared-off and groove spine showed no asymmetric spreading (Fig. S5b,c) and didn’t move (Fig. S6a,b). From these experiments we deduce that the scales and the splayed capillary tubes formed by the scales on the spine are most possibly responsible for the directional and spontaneous motion of the first droplet.

We further deposited a second drop on the scale-covered spine to find the reason for the relative fast movement of fog drops 3′ and 4′ (Fig. 4a). After the movement of the first drop from the top to bottom of spine at ~152 s, the second droplet acts fast subsequently. As shown in Fig. 4a, the second deposited drop (drop 2) took about 0.07 s to move the same distance. The different moving velocities of these drops can be estimated by the slope degrees of the lines which represent the relationship between the drop moving distance and the moving time (as showed in Fig. 4b). The drop 1 moves with a velocity ~0.03 mm/s and drop 2 moves with a velocity ~70 mm/s. The high speed video helped us to observe the moving activities of the second drop in detail (see Fig. S7). The second drop firstly moved towards the first drop stably (from 0.000 s to 0.030 s) and then coalesced with it (from 0.036 s to 0.058 s). The fast motion of droplet before coalescence could rule out the coalescence influence on droplet movement. In addition, we found after the passing of drop 1, there would remains ultra thin water film on the spine (Fig. 4a, from the time ~20 s to ~120 s, see the white bright area on the surface of the spine). According to the above observations, we conclude the water ultra thin liquid film may contribute to the fast transport of the subsequential droplet.

Base on the above observations, we give explanations for the directional droplet motion on scale-covered spine. Figure 5 is used to illustrate the behaviors of part of droplet liquid on the spine. The other parts of the droplet liquid exhibit the similar behaviors because the spine is symmetric in cross section. It is understood that when a drop firstly forms on the spine, its shape is distorted by the asymmetric structure of the scales (Fig. 5b). The drop tends to spread along the scale due to the disparity between the local contact angle of the drop on the spine and the nascent contact angle of the related drop end, thus the initial spreading of the drop is triggered^26^. Influenced by the asymmetric scale-by-scale, the contact angle of the right side of the drop (*θ*_*r*_) on the spine is larger than the contact angle of the left side of the drop on the spine (*θ*_*l*_) (the inset of Fig. 5b, frame b1). A driving force *F*_*d*_ is described as follow^27^,^28^:


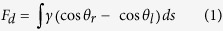


where *θ*_*r*_ is the contact angle of the right side of the drop, *θ*_*l*_ is the contact angle of the left side of the drop, *γ* is the liquid-solid surface tension and d*s* is the integrating variety along the length from the right to the left side of the drop. Because *θ*_*r*_ > *θ*_*l*_, *F*_*d*_ points to the bottom of the spine and it propels the drop move to the left.

Only considered into the fiber with normal scale-by-scale along its axis, drop motion would cease when the drop reaches the equilibrium configuration, e.g., the ends of drops can be “hinged” by the edges of scales^29^. Interestingly, the scales on spine of cactus g*ymnocalycium baldianum* open separately. The stable conformation of the drop will be disturbed by the liquid flow in the space among the tilted-up scales^30^,^31^,^32^. When the drop reaches the hinge of scale 2 (on the left side of the droplet) and scale 0 (on the right side of the droplet), the liquid on the right and left sides of the drop could continue to spread along the direction parallel to the axis of spine. Because of the asymmetric radius from the bottom and the top cavities of the scales (inset of Fig. 5b, frame b2, *r*_*c*_ ~8 μm < *R*_*c*_ ~20 μm), the spaces of cavities can be regarded as typical splayed capillary micro-tubes in array. In the spreading process, the liquid of the right side of the drop firstly enters into the bottom of the scale 0 and exhibit a concave curvature owing to the hydrophilicity of the scale surface. The Laplace pressure resulted from this curvature is defined as *P*_*0*_, which points to the tip of the scale (inset of Fig. 5b, frame b3). *P*_*0*_ will drag the droplet liquid move to the left of the spine. Influenced by the hinge scales 0 and 2, the main liquid of the drop exhibits a nearly symmetrical shape, so the Laplace pressure (*P*_*1*_) resulted from the convex drop curvature points vertically to the axis of spine, which have no influence on droplet motion in horizontal direction. The liquid of the left side of the drop exhibits concave curvatures when it contacts with scale 3. The Laplace pressures introduced by the meniscuses at top and bottom of scale 3 are defined as *P*_*2*_ and *P*_*3*_, respectively. *P*_*2*_ points to the top of the scale and *P*_*3*_ points to the bottom of the scale. Both of *P*_*2*_ and *P*_*3*_ will drag more droplet liquid into scale 3, in a way, they will help the more droplet liquid move to the left (inset of Fig. 5b, frame b4). Therefore, according to the different dragging effect applied on the whole droplet, the Laplace pressure difference for the entire drop can be described as 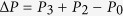. 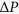 can be further written as 
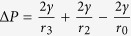
, where *r*_*0*_, *r*_*2*_ and *r*_*3*_ are the curvature radii of the related liquid menisci, respectively. As for a liquid menisci in a capillary tube with a radii of *R*, its curvature radii *r* can be written as 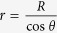, *θ* is the contacting angle of the liquid on the capillary tube surface. Based on this equation it can be deduced *r* decreases with *R*. The curvature radii difference between the meniscuses at bottom and top of liquid leads to the difference between *P*_*2*_ and *P*_*3*,_which generates a force pointing to location of the micro-tube with minimum radius^33^,^34^. So the liquid of drop reaches the micro-tube end firstly, and then extends along the scale. From this aspect the bottom liquid menisci in scale 3 will keep the smallest curvature radius *r*_*3*_ because the liquid has reached the bottom of the scale. *r*_*0*_ will grow large as more liquid has entered into the space under scale 0. So with the liquid spreading, *r*_*3*_ > *r*_*0*_and 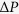 > 0. 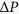 points to the left side of the drop and will drive the drop liquid to move left. When the droplet liquid totally fills the space between scale 2 and scale 3, this part of droplet liquid will merge with the main droplet. Thus the hinge effect of scale 2 will be overcome and the whole droplet will move to the left (Fig. 5c). This hypothesis is supported by the observed phenomenon that the drop showed a obvious movement when the space between scale 1 and scale 2 was filled (Fig. 3b, from 20.26 s to 20.27 s). The drop also could overcome the hinge effect of the scale 3 by the same way (Fig. 5d). The droplet thereby moves continuously along the scale-by-scale array surface of the spine.

The subsequent drops with fast moving velocity are mostly attributed to the ultra thin liquid film on the scale-covered spine left by the first drop. In fog atmosphere, the direction of the fog flow is vertical to the spine, water will form on the surface of the tiled scale and not easily form under the scale, so in high humidity the channels are not totally wetted and the first droplet encounters high surface adhesion. After the past of the first droplet, the splayed capillary micro-tube makes part of the drop liquid trapped in it by capillary force, thus all the liquid trapped in the capillary micro-tube arrays forms a liquid film on spine surface benefiting from the discontinuous scales (Fig. 5d). The liquid film leads to a more hydrophilic spine surface, which greatly decreases the surface contact angle hysteresis^35^,^36^,^37^. When the subsequent drop is formed on the surface, the unbalanced surface tension force produced from the contacting angle difference between the opposite sides of the drops and the Laplace pressure difference resulting from the liquid curvature disparity cooperatively initiate the directional drop transport. Benefiting from the low friction force, the sequent drop could move more quickly. To confirm the lubricant effect of the liquid film, we imbued the bottom part of the spine in water and found the drop moves much fast on liquid-film area (see Fig. S8), which is a strong support for our theory. Directional droplet transport could renew the surface effectviely^19^ and this is beneficial for the continuous formation of droplets, hence materials with fast directional droplet transport ability would have a high efficiency in fog collection.

In summary, a kind of scale-covered cactus spine could transport droplet spontaneously and quickly. The asymmetric splayed capillary channel arrays formed by the scales on the spine surface provide the initial propulsion for the drop motion. The trapped liquid left by the first droplet in the splayed tubes contributes to the low friction force of the surface, and then the following droplets can move more quickly. Our works find a novel way to transport droplets spontaneously and quickly with liquid trapped in the surface structure acting as the lubricant. This finding is helpful to design artificial cactus spines to develop a fast water transport system without additional energy. It also provides us with an applicable way to prompt the liquid-transferring efficiency on interfaces of materials^38^.

## Additional Information

**How to cite this article**: Liu, C. *et al.* Effective directional self-gathering of drops on spine of cactus with splayed capillary arrays. *Sci. Rep.* **5**, 17757; doi: 10.1038/srep17757 (2015).

## Supplementary Material

Video S1

Video S2

Supplementary Information

## Figures and Tables

**Figure 1:**
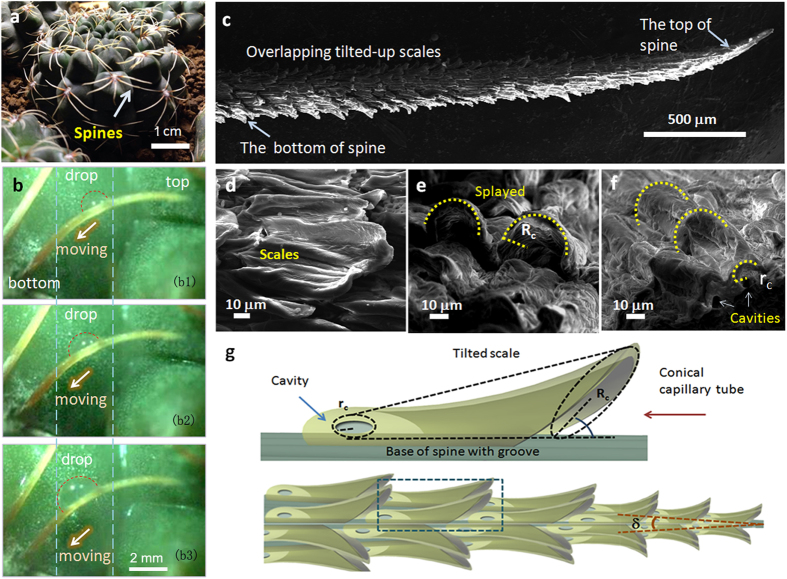
(**a**) Photo of *Gymnocalycium baldianum* cactus with clusters of 5–6 spines. (**b**) Optical observation of condensed drop moving on spine (Frame b1-b3). (**c-f**), ESEM images of the spine. The surface of the spine is covered with scales, and the tips of the scales tilt at an angle (*α*) of ~20°. Cone spine opens an apex-angle *δ* of ~15° (**c**). Each scale has a width of ~30–50 μm and a length of ~100–130 μm (**d**). The tilt-up tips of the scales open a semicircle with a radius (*R*_*c*_) of ~20 μm (**e**). The cavities are formed between the scales and the surface of the spine with a radius (*r*_*c*_) of ~8 μm (**f**). (**g**) Illustration for the scale-covered spine with an apex-angle *δ*. The space between the scale and the surface of the spine forms a splayed capillary channel, opening different radius of *R*_*c*_ and *r*_*c*_ in two ends. Under the scale, there are parallel grooves.

**Figure 2:**
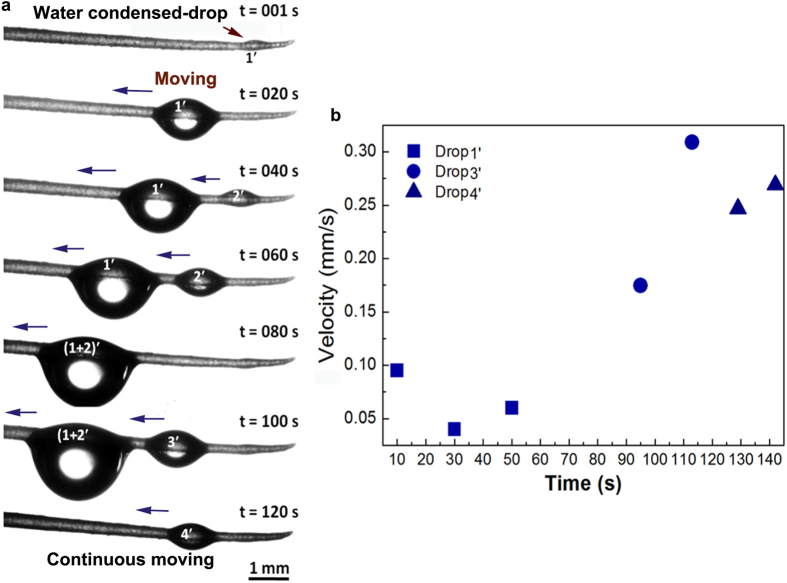
(**a**) Fog water collecting phenomenon of the scale-covered spine. Drop 1′ formed on the spine and moved to the bottom of the spine. Subsequently drop 2′ formed and coalesced with drop 1′ and drop (1 + 2)′ was obtained. After the formation of drop (1 + 2)′, drop 3′ and drop 4′ formed in turn and then both of them moved to the bottom of the spine. (**b**) The moving velocities of the fog drops 1′, 3′ and 4′ without influence of coalescence. Both of drops 3′ and 4′ moved more quickly than drop 1′.

**Figure 3:**
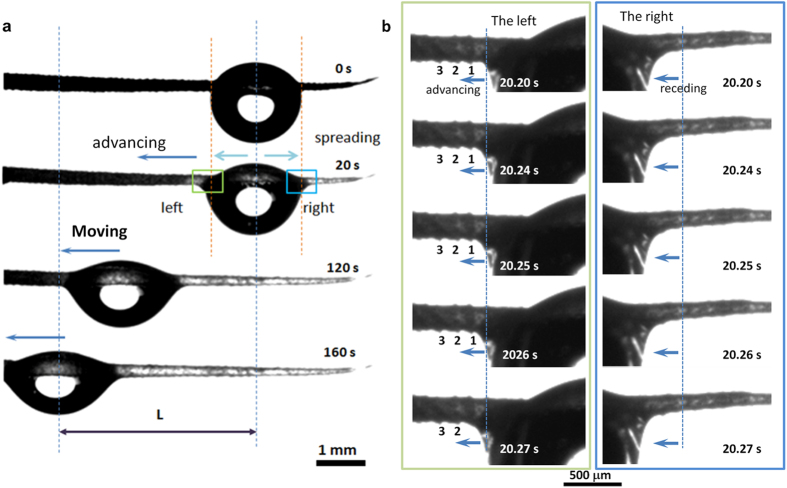
(**a**) Drop transport on the scale-covered spine. A drop (2 μl) was deposited on horizontal spine. After ~20 s, the drop spread asymmetrically along the spine. Subsequently, the drop moved directionally at ~120 s. After ~160 s, drop almost moved along the spine. (**b**) *In-situ* observation of drop moved from one scale to another (from ~20.20 s to ~20.27 s). The left-side images show the liquid of the drop moved along the scales and advancing to cover over scale 1 at ~20.27 s. The right-side images show the right-hand of the drop moved along the scales, receding to show the scale at ~20.27 s.

**Figure 4:**
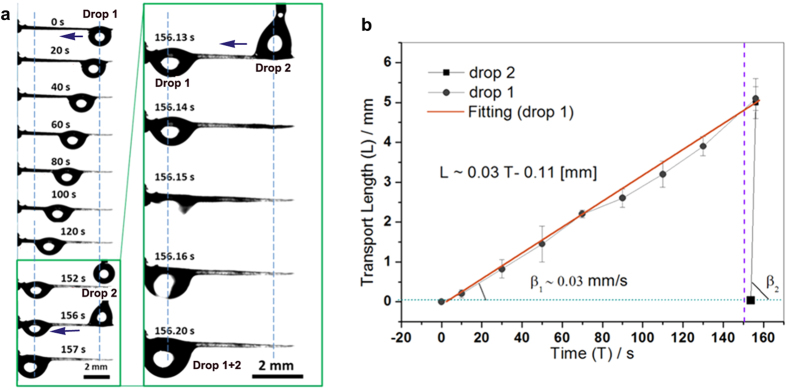
Continuous self-transport of the drops on the surface of spine. (**a**) Continuous self-transport of drops on spine. The subsequent drop 2 moved fast in the time from ~156 s to ~157 s after the initial drop 1 passed over the surface of spine in time of ~1–152 s. The insets show the process of subsequent drop 2 that moved fast to the bottom of the spine in ~156.14–156.20 s. (**b**) Moving distances versus time of the initial drop (drop 1) and subsequent drop (drop 2). The average velocity of drop 1 can be estimated with β_1_, that of drop 2 is β_2_ (β_2_ > β_1_), indicating drop 2 moved much fast than drop 1.

**Figure 5:**
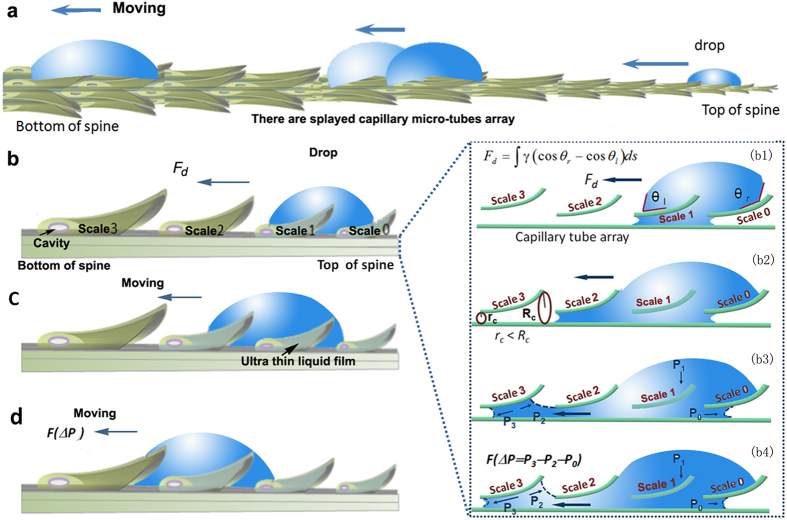
Illustration of splayed capillary micro-tubes induced self-transport of drop. (**a**) Drop moving continuously on the spine with splayed capillary micro-tubes array composed of overlapping titled-up scales from the top to bottom of spine as an integrating regime for water self-gathering. (**b**) The effect of scale-by-scale array and formation of ultra low adhesion. As the drop is condensed on the initial scale defined as scale 0 at top of spine. The insets (Frames b1-b4) indicate the effect of capillary micro-tube array on spine for drop self-transport. Drop spreads asymmetrically as a result of the different contact angles on the left (*θ*_*l*_) and right (*θ*_*r*_) sides (Frame b1). The driving force (*F*_*d*_) can be generated by the difference of advancing (left) contact angle and receding (right) contact angle. Drop moves directionally influenced by the splayed capillary micro-tubes. Liquid meniscus in scale 0 introduces Laplace pressure *P*_*0*_, which propels the drop liquid into scale 0 and spread over location of scale 1. *P*_*0*_ decreases with the growing liquid menisci in scale 0 (Frame b2). The liquid of the left side of the drop enters into the capillary micro-tube formed by scale 2. The Laplace pressure (*P*_*2*_ and *P*_*3*_) introduced by the curvatures of liquid in scale 2 drags the liquid tend to move to location of the scale 3 (Frame b3). *P*_*3*_ is growing larger with liquid spreading and finally will be larger than *P*_*0*_. When the Laplace pressure difference Δ*P* produced by cooperation of *P*_*0*_, *P*_*2*_ and *P*_*3*_ is large enough, the entire drop overcomes the hinge effect of scale 2 and move toward location of scale 3 (Frame b4). (**c,d**) Drop self-transport from scale-by-scale and formation of ultra-thin film over surface of spine. Drop moves directionally due to driving force resulted from the asymmetric contact at interface of scale-by-scale (**c**). Due to the cavities between splayed scales and groove base of spine, the drop remains the ultra thin liquid film along overlapping scales to form ultra low-adhesive surface on spine (**d**).
