# Supplementary material for: Effective directional self-gathering of drops on spine of cactus with splayed capillary arrays
Source: Sci Rep. 2015 Dec 7;5:17757. doi: 10.1038/srep17757 (PMC4671016; doi:10.1038/srep17757)

Effective directional self-gathering of drops on spine of cactus with splayed capillary arrays

Chengcheng Liu, Yan Xue, Yuan Chen, Yongmei Zheng*

Key Laboratory of Bio-Inspired Smart Interfacial Science and Technology of Ministry of Education, School of Chemistry and Environment, Beihang University, Beijing, 100191 (P. R. China).
*Corresponding author: e-mail: zhengym@buaa.edu.cn

Content：

Supplementary Methods
Supplementary Figure Legends： Figure S1-S8

SUPPLEMENTARY METHODS

Methods:

Sample preparation:

All cactuses were purchased from local flower store, Beijing, China. The samples of spines were picked from the ball and cleaned with deionized water. Then the spines were dried at the ambient environment before experiments. The samples of clear-off-scaled spines were prepared by using sand paper to abrade scales, rinsed by deionized water, and then were dried at the room temperature.

Characterization:

The microstructures of spines were observed by the environmental scanning electron microscopy (ESEM, Quanta FEG 250, FEI, America) with a low vacuum mode and the accelerating voltage is 10 kV. Contact angles of water were tested by optical contact angle meter system (OCA Micro40, DataPhysics, Germany). Dynamic processes of water transport were recorded by CCD camera in optical contact angle meter system. High-resolution processes of droplet movement were observed by high speed video (at a speed of 1000 frame per second) at ambient temperature. Drops of 2.0 μl in volume were used in experiments. The samples of spines were clamped by the metal stage.

Experiment of fog drops transportation:

A sample of cactus spine was exposed to fog flow composed of microscale water beads generated by an ultrasonic humidifier (SC-4317, Beijing Yadu Science and Technology Co., China). The fog flow was generated at a rate of 30 cm/s, and the experiment temperature was 20°C. The outlet of the fog flow was put diagonally above the sample. An optical contact angle meter system with a CCD camera connected to desktop computer was used to record the behaviors of fog drops on the spine. High-speed video (Phantom V9.1, Vision Research, USA) was used to observe two drops coalescing on the spine.

SUPPLEMENTARY FIGURE LEGENDS

Figure S1 Fog water collecting phenomenon of tilted spine. Fog drops moved towards to the bottom of the spine.

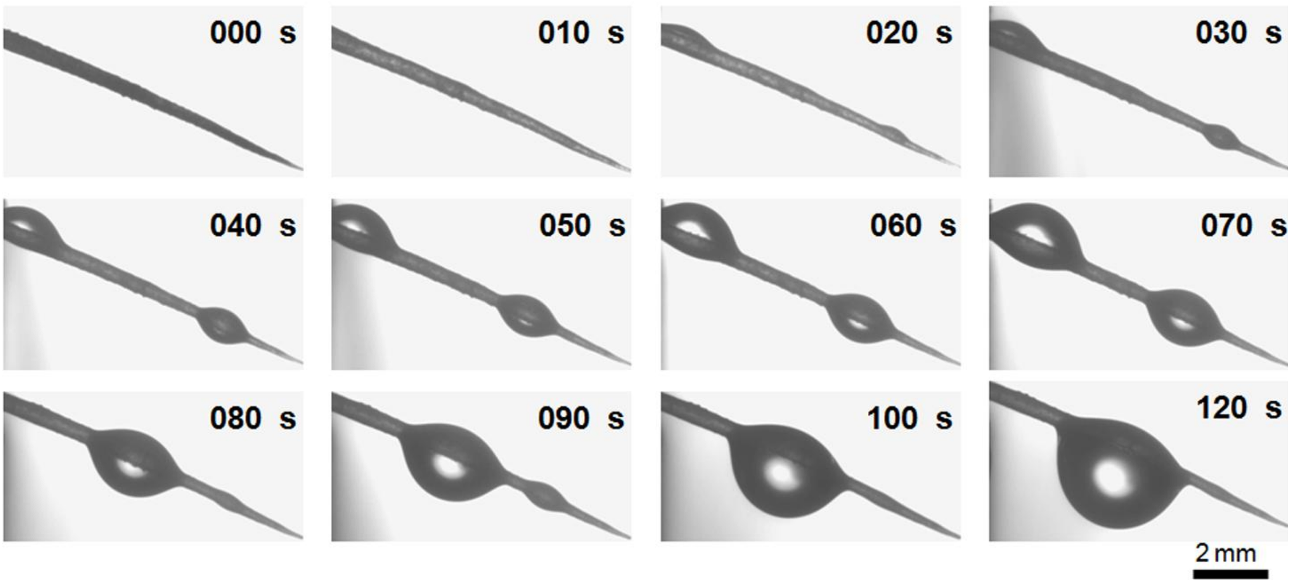

Figure S2. Compare between advancing and receding contact angles. The contacting angles measurements reveals there is no obvious difference between the contacting angles of the left and right sides of the drop. It implies that droplet spreads the liquid along the surface of spine with scale-by-scale due to capillary.

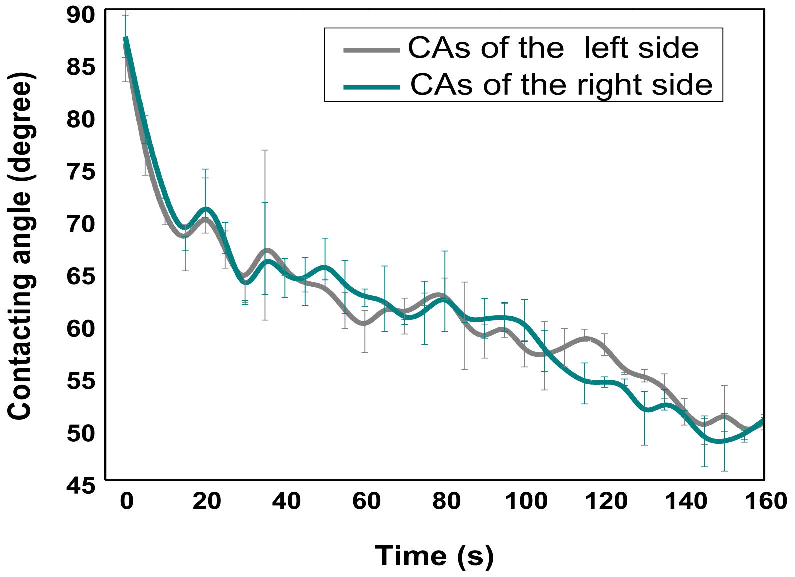

Figure S3 | ESEM images of scale-cleared-off spine. Spine with scales cleared off. Magnified image shows rough surface of scale-cleared-off spine.

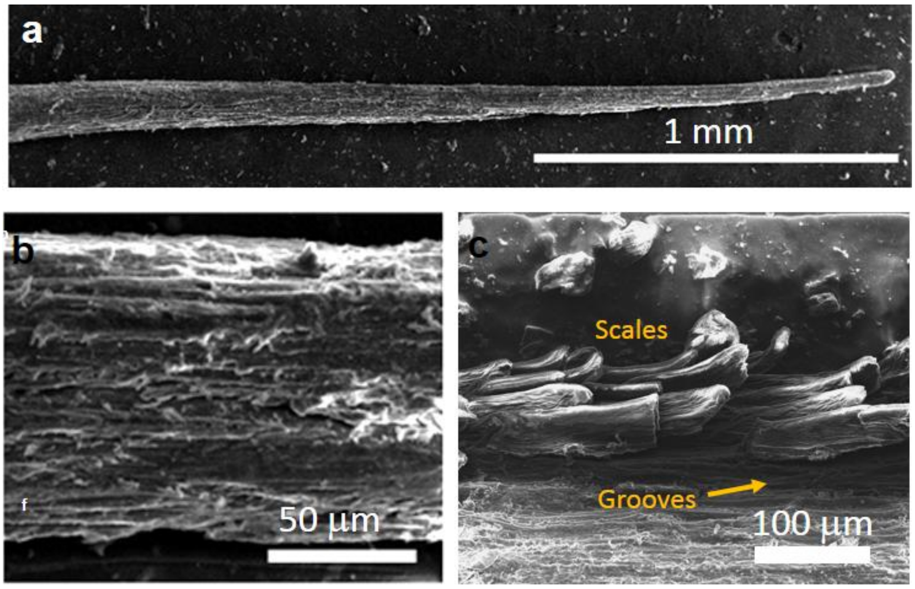

Figure S4 | a-c. Photograph of cactus with groove-covered spines (a), ESEM image shows the groove-covered spine (b), Magnified image show the groove-covered spine with groove periods range from ~ 10–20 μm (c).

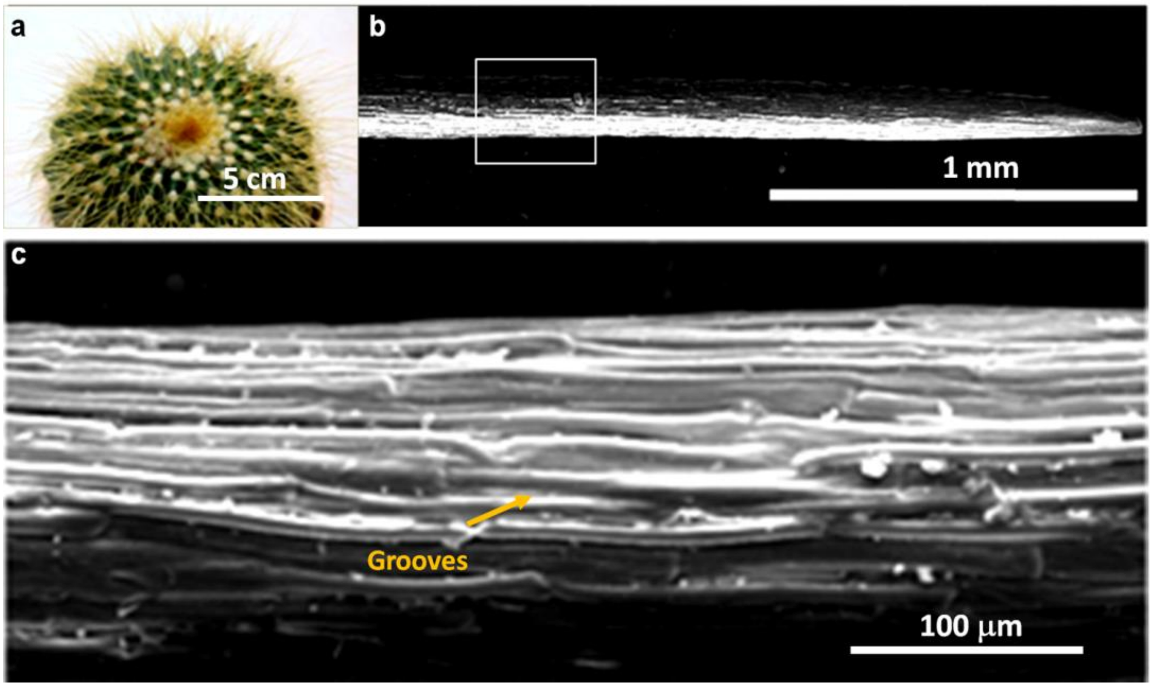

Figure S5 | Initial states of drops deposited on different surfaces of spines. a, A drop deposited on the scale-covered spine. The drop showed an inclination to move to the bottom of the spine. **b**, A drop deposited on the scales-cleared-off spine. The drop showed no asymmetrical extension. **c**, A drop deposited on the groove-covered spine. The drop sat on the surface of the spine and showed no expanded activity.

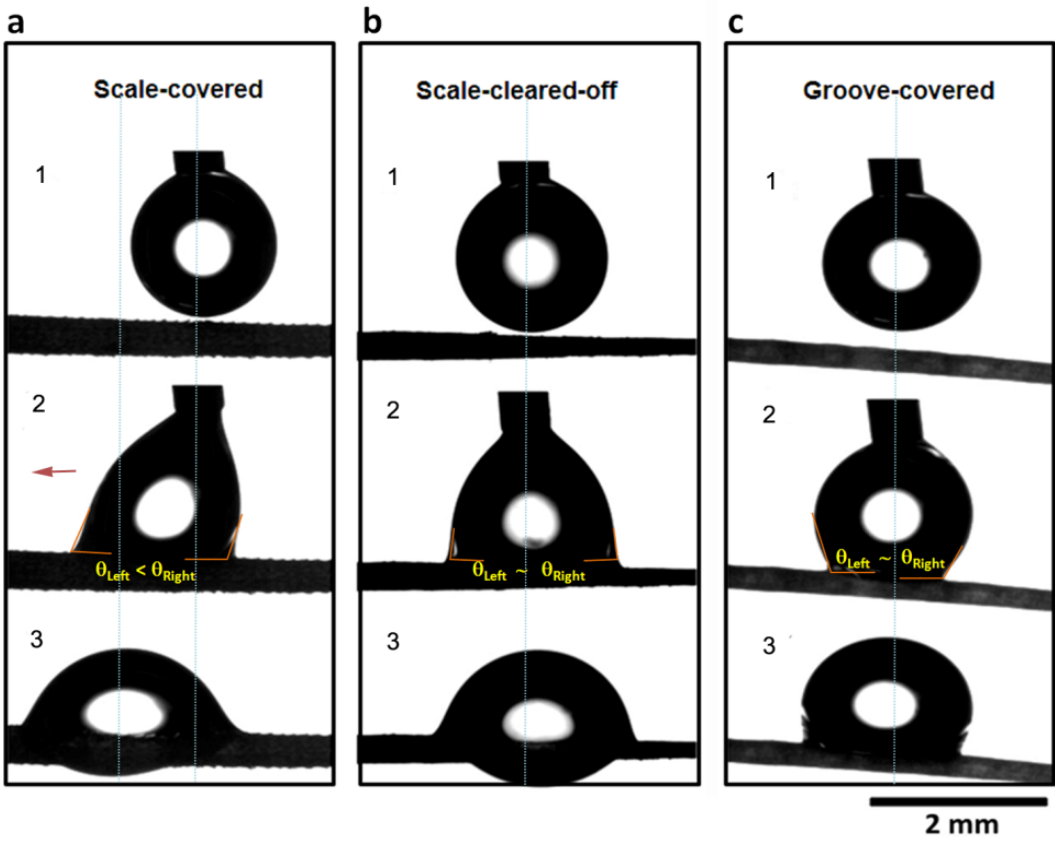

Figure S6 | Drop transport abilities on spines with different surface structures. **a**. A drop deposited on the scale-cleared-off spine. The drop just expanded but exhibited no moving activity. **b**. A drop deposited on the groove-covered spine. The drop just hung on the spine and exhibited no moving activity. The drop is ~ 2 μl in volume.

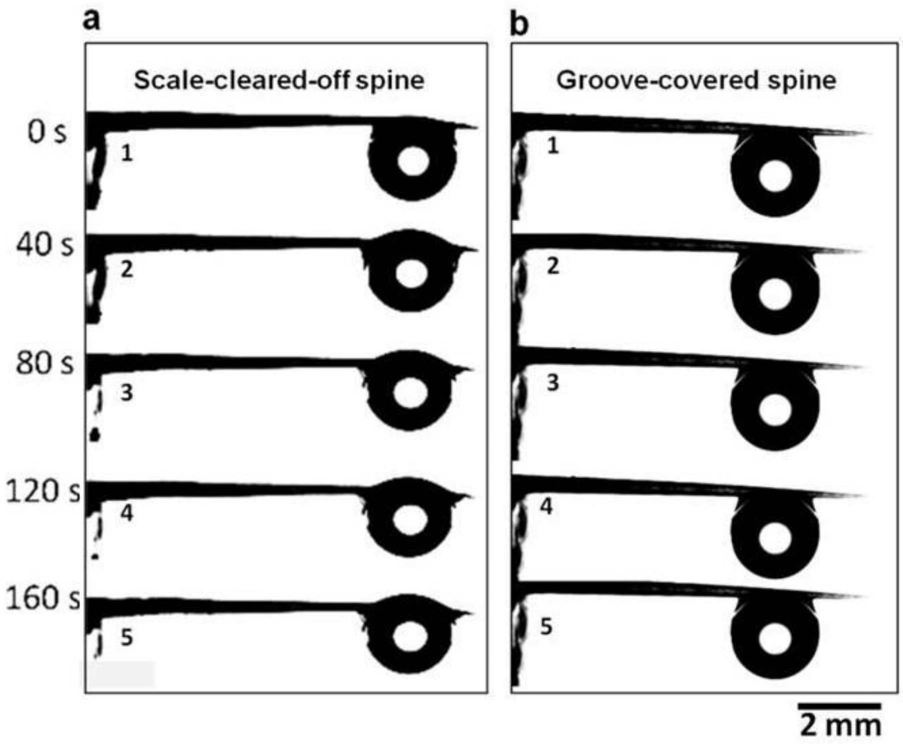

Figure S7 | High speed video images of drop behaviors on surface of cactus spine. When the second drop was deposited on the spine surface, it firstly moved directionally to the first drop (from 0.000 s to 0.030 s). Then it coalesced when the first drop. The coalesced drop continued to move directionally to the bottom of the spine (from 0.039 s to 0.058 s).

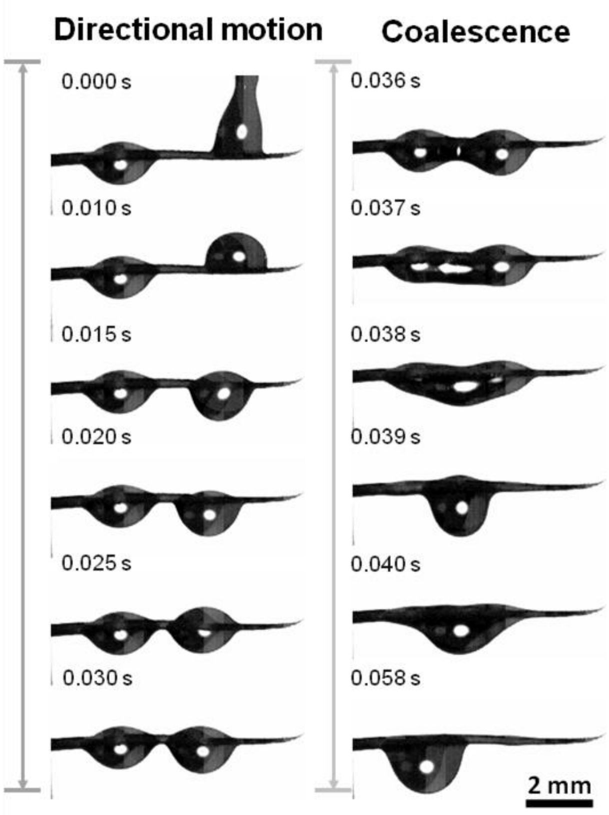

Figure S8 | Drop movement on the spine of cactus *Gymnocalycium baldianum*. (a) Pretreated spine with liquid film formed from the middle to bottom of spine. The vertical line represents the board line of the water film area and no water film area. **(b)** Moving velocity of the drop on the both regions with water film and without water film. Drop moved more quickly in the water film area.

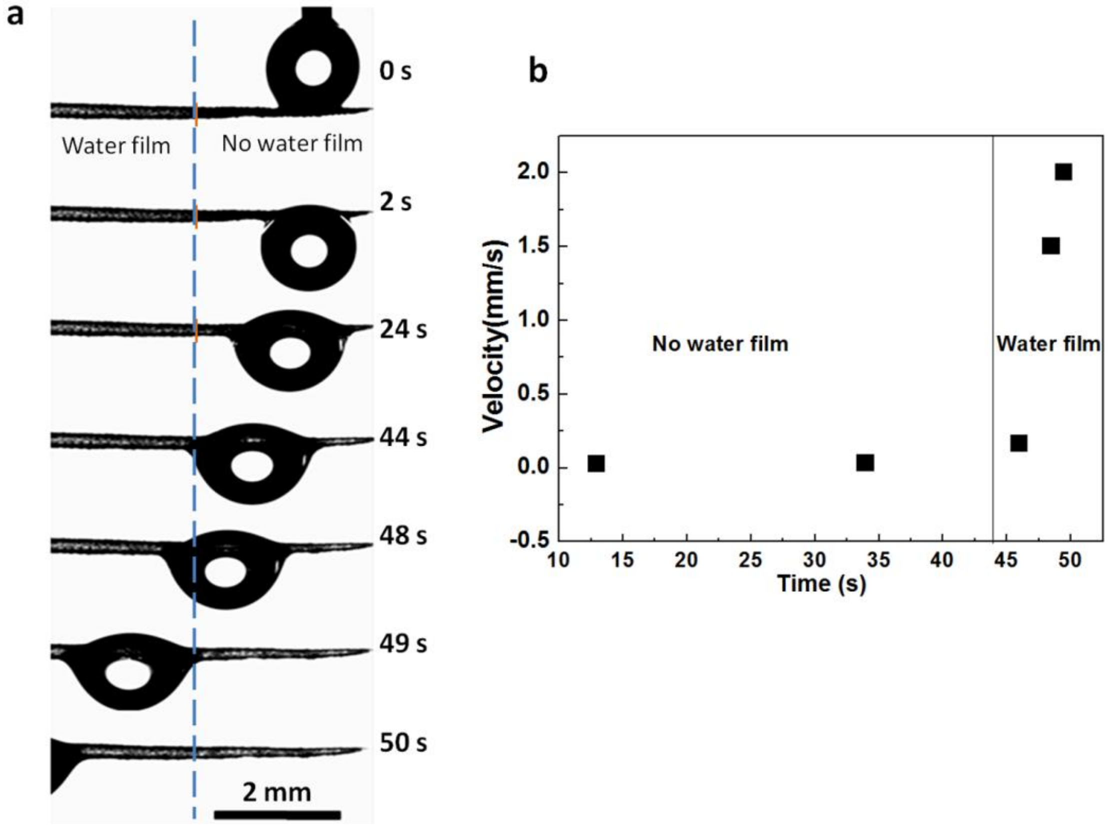

Supplement: Supplementary Information [file srep17757-s3.pdf]
